# Supplementary material for: The Food Environment of Primary School Learners in a Low-to-Middle-Income Area in Cape Town, South Africa
Source: Nutrients. 2021 Jun 15;13(6):2043. doi: 10.3390/nu13062043 (PMC8232268; doi:10.3390/nu13062043)
Supplement: Supplementary file 1 [file nutrients-13-02043-s001.zip › nutrients-1227666-supplementary.pdf]

Table S1: School principal interview guide

	**Primary question**	**Prompts**
1	Can you describe the learner profile in the school – areas where they come from etc.?	• Why are children bussed in from other areas -is it a department of health specification?<br>• Can you elaborate on the learners' home circumstances?
2	How healthy do you think the learners in your school are?	• Elaborate on any particular problems<br>• Reasons for these problems?
3	Can you describe your personal philosophy regarding what a health promoting school should involve?	• What are the initiatives that you have personally implemented/promoted to further your philosophy?
4	Can you summarize to what extent parents are involved in health promoting activities at school?	• What effect do you think this can have/has on the health of the learners
5	What type of health screening of learners does the Department of Health/Basic Education offer/promote in your school?	• What can the school do to ensure that this screening takes place on a regular basis?
6	Which formal health promotion activities does the Dept of Basic Education expect schools to run?	• Are these activities run by your school?<br>• Are they effective?<br>• How are parents involved in these types of initiatives?
7	To what extent is the health promotion component of the life-orientation curriculum taught in your school?	• How do educators teach the nutrition content?
8	How do you experience the roll out of the school feeding scheme at your school?	• Any concerns re the menu, delivery of foods to your school, preparation and serving of the food, feeding time, quality of food, coverage of the needy learners and satisfaction of learners with the food
9	How healthy do you think the foods/snacks are that learners in your school eat?	• Any particular concerns?<br>• Home vs tuck-shop vs vendors<br>• Reasons for particular eating food choices/eating habits?
10	To what extent do you believe school polices around what is sold at the tuck-shop can contribute to helping children to make healthier food choices?	• Any particular policies that are in place in your school?<br>• National of provincial?<br>• Some not implemented?
11	Who runs the tuck-shop at the school?	• How is it run?<br>• Towards what is the profit of the tuck shop being used?

		• how important is this profit to the school?
12	Does the sale of foods or snacks form part of fundraising events or activities?	• if so, which types of events<br>• what types of foods?
13	To what extent do you believe school polices around what is sold at school events (social and sport events) can contribute to helping children make healthier food choices?	
14	To what extent do you believe school policies around what is served at staff functions can contribute to helping children make healthier food choices?	
15	To what extent do you think educators influence what children choose to eat or drink?	• Responsibility of the school for the diet of students?<br>• The degree of priority given to food and nutrition?<br>• Educators supportive of healthy eating?
16	Anything else you would like to mention?	

Table S2: Inductively derived thematic framework of the key informant interview with the school principal.

Theme	Sub-theme	Sub-sub-theme	Sub-sub-sub-subtheme
School	School feeding	Influence on what is given	Fortified drink
	Vegetable garden	Usage	School meals
	Tuckshop	Unhealthy option	Selling healthy items
	Teachers role modelling	Eating in front of learners	Types of healthy vs unhealthy foods
Community	Neighbourhoods	Unsafe	Purchasing behviour
	Food outlets	Food vendors on route to school e.g. sidewalk vendors	Items sold: Traditional foods
		Food vendors on route to school e.g. sidewalk vendors	Items sold: Cheap, unhealthy food - chips, sweets…
		Vendors around school premises	Types of food sold
		Control over what vendors sell	Provide in needs of some children
Home	Socio-economics	Informal housing	Cost of food
	Food availability	Little or no food at home	Hunger
	Family structure	Parents	Struggles in life

Figure S1: Photovoice thematic framework-code list and frequency of mentions

Code list

Theme	Sub-Theme	Sub-subtheme	Sub-sub-subtheme	Code
Where buy	Vendors	What	Groceries (maize meal, spices, bread, etc.	1111
			Fruit	1112
			Veg	1113
			Snacks and Colddrink	1114
		Location	Close	1121
			Not so close	1122
		How often purchases made	Daily	1131
			Not daily	1132
	Supermarkets	What	Groceries (maize meal, spices, bread, etc.	1211
			Fruit	1212
			Veg	1213
			Snacks and Colddrink	1214
	Side walk food/snacks	What	Chicken Feet	1311
			Gizzards	1312
		Location	Close	1321
			Not so close	1322
		How often purchases made	Daily	1333
			Not daily	1331
	Food outlet	What	Fish and chips	1411
			Russians	1412
			Vetkoek	1413
			Kota (bread with chips)	1414
			Burgers	1415
		Location	Close	1421
			Not so close	1422
		How often purchases made	Daily	1431
			Not daily	1432
	Mother's work	What	Snacks (chips, popcorn, sweets etc.)	1511

			Groceries (maize meal, spices, bread, etc.	1512
		How often items are brought home	Daily	1521
			not daily	1522
	School tuckshop	What	Snacks (chips, popcorn, sweets etc.)	1611
		How often purchases made	Daily	1621
			Not daily	1622
What's available at home	Protein	Red meat		2110
		Chicken		2120
		Fish		2130
		Legumes		2140
		Eggs		2150
		Processed meat	Polony	2161
			Russians	2162
	Starch	Starchy Veg (Potatoes, mielies, butternut)	Potatoes	2211
			Mielies	2212
			Butternut	2213
		Refined starch	Maize meal	2221
			White rice	2222
			white bread	2223
			Pasta	2224
		High fiber	Brown bread	2231
			Weetbix	2232
	Vegetables	Common	Onion	2311
			Tomato	2312
			Carrots	2313
			Cabbage	2325
		Uncommon	Spinach	2321
			Covo	2322
			Broccoli	2323
			Cauliflower	2324
			Peppers (Capsicums)	2326
			Other	2327
		How often eaten	Daily	2331
			Not daily	2332
	Fruit	Common	Apples	2411

			Oranges	2412
			Bananas	2413
		Uncommon	Berries	2421
			Pineapple	2422
			Other	2423
		How often eaten	Daily	2431
			Not daily	2432
	Fats	Mayonnaise		2510
		Margarine		2520
		Oil		2530
		Peanutbutter		2540
	Snacks	Chips		2610
		Sweets and chocolates		2620
		Cakes and muffins		2640
		Other (Chicken feet, slangetjies, nuts)		2630
	Beverages	Fizzy drinks		2710
		Fruit juice		2720
		Concentrate mixed with water		2730
	Dairy	Milk		2810
		Cheese		2820
Meal Compostion	Protein + veg + starch	Protein	Chicken (with skin)	3111
			Red meat	3112
			Legumes	3113
			Fish	3114
			Soya	3115
			Eggs	3116
		Veg	Carrots	3121
			Onion + tomato	3122
			Spinach/Covo	3123
			Broccoli	3124
			Cauliflower	3125
			Salad (Beetroot, green salad)	3126
			Other (e.g. Cabbage, peppers)	3127
		Starch	Potatoes (as part of stew or potato salad)	3131
			Mielies	3132
			Butternut	3133
			Maize meal	3134

			Rice	3135
			Pasta	3136
	Protein + majorly starch	Protein	Chicken	3211
			Red meat	3212
			Legumes	3213
			Fish	3214
			Soya	3215
			Eggs	3216
			Processed meat	3217
		Starch	Potatoes (as part of stew or potato salad)	3221
			Mielies	3222
			Butternut	3223
			Maize meal	3224
			Rice	3225
			Pasta	3226
			Samp	3227
			Bread	3228
	Bread meals	Bread	White	3311
			Brown	3312
		Fillings	Polony	3321
			Russians	3322
			Peanut butter	3323
			Burger patty	3324
			Egg	3325
	Special meals	Sundays		3410
		Funerals		3420
		Birthdays		3430
	Only Starch	Hot chips + bread		3510
		Hot chips		3520
		Maize meal		3530
		Rice + potatoes		3540
Family	Eating together	Daily		4110
		Not daily		4120
	Where they eat	In front of the TV		4210
		At the table		4220
		In the bedroom		4230
	What they eat together	Meals		4310
		Snacks	Chips	4321
			Sweets	4322

			Other (Chicken feet, slangetjies)	4323
Peer engagement	Eating together	Daily		5110
		Not daily		5120
	What they eat together	Take aways		5210
		Snacks	Chips	5221
			Sweets	5222
			Fizzy drinks	5223
		Sandwiches		5230
	Where they eat	At school		5310
		At each others' houses		5320
Likes	In family	Fruit		6110
		Vegetables		6120
		Meals		6130
		Snacks	Chips	6141
			Sweets and chocolates	6142
			Other (cake, chicken gizzards)	6143
		Fizzy drinks		6150
	In friends	Snacks	Chips	6211
			Sweets	6212
			Other (chicken feet, slangetjies)	6213
		Fizzy drinks		6220
Dislikes	In family	Fruit		7110
		Veg		7120
		Snacks	Chips	7130
			Sweets	7132
			Other (chicken feet, slangetjies)	7133
	In friends	Fruit		7210
		Veg		7220
		Snacks	Chips	7230
			Sweets	
			Other (chicken feet, slangetjies)	
Food Preparation	Who prepares meals	Mother		8110
		Father		8120
		Learner		8130
		Other (Friend's mum, aunty, grandmother ..)		8140
	Equipment	Two-plate stove		8210
		Gas stove		8220

		Braai		8230
		Paraffin heater		8240
	Healthy	Stir frying		8310
		Braai		8320
		Stews/Curry		8330
	Unhealthy	Frying		8410

Frequency of mentions

Theme 1: Where buy

S-theme	SS -theme	SSS - theme	Count
Vendors			
	What	Groceries (maize meal, spices, bread, etc.	12
		Fruit	8
		Veg	5
		Snacks and Colddrink	16
	Location	Close	13
	How often purchase made	Daily	7
		Not daily	3
**Total Counts**			**64**
Supermarkets			
	What	Groceries (maize meal, spices, bread, etc.	10
		Fruit	1
		Veg	1
		Snacks and Colddrink	8
**Total Counts**			**20**
Side-walk			
	What	Chicken feet	1
		Gizzards	1
	Location	Close	1
	How often purchases made	Not daily	1
**Total Counts**			**4**
Food outlet			1
	What	Fish and chips	3
		Russians	2
		Vetkoek	1

		Kota (bread with chips)	2
		Burgers	1
	Location	Close	3
	How often purchases made	Daily	1
		Not daily	3
**Total Counts**			**17**
Mother's work			
	What	Snacks (chips, popcorn, sweets etc.)	5
		Groceries (maize meal, spices, bread, etc.	2
	How often items are brought home	Daily	1
		not daily	3
**Total Counts**			**11**
School Tuck-shop			
	What	Snacks (chips, popcorn, sweets etc.)	3
	How often purchases made	Daily	1
**Total Counts**			**4**

Theme 2: What's available in the home

**S-theme**	**SS -theme**	**SSS - theme**	**Count**
Protien			
	Meat		15
	Chicken		14
	Fish		6
	Legumes		7
	Eggs		4
	Processed meat	Polony	9
		Russians	3
**Total Count**			**58**
Starch			
	Starchy veg	Potatoes	20
		Mielies	5
	Refined starch	Maize meal	13
		White rice	9
		white bread	15
		Pasta	5

	High fiber	Brown bread	2
		Weetbix	1
	**Total count**		**70**
Vegetables			
	Common	Onion	14
		Tomato	17
		Carrots	10
		Cabbage	13
	Uncommon	Spinach	4
		Covo	3
		Broccoli	2
		Cauliflower	1
		Peppers (Capsicums)	8
		Other	3
	How often eaten	Daily	6
		Not daily	7
	**Total Count**		**88**
Fruit			
	Common	Apples	4
		Oranges	2
		Bananas	3
	Uncommon	Berries	2
		Pineapple	1
		Other	1
	How often eaten	Daily	1
		Not daily	2
	**Total Count**		**16**
Fats			
	Mayonnaise		8
	Margarine		5
	Oil		6
	Peanutbutter		3
	**Total Count**		**22**
Snacks	Chips		6
	Sweets and chocolates		5
	Cakes and muffins		4
	Other		2
	**Total Count**		**17**
Beverages			
	Fizzy drink		6

	Fruit Juice		3
	Concentrate mixed with water		2
**Total Count**			**11**
Dairy			
	Milk		5
	Cheese		2
**Total Count**			**7**

Theme 3: Meal composition

**S-theme**	**SS-theme**	**SSS- theme**	**Count**
Protein + veg + starch			
	Protein	Chicken	8
		Red meat	11
		Legumes	2
		Fish	5
		Soya	2
		Eggs	2
**Sub -Total Count**			**30**
	Veg	Carrots	7
		Onion + tomato	15
		Spinach/Covo	6
		Broccoli	2
		Cauliflower	1
		Salad (Beetroot, green salad)	11
		Other (e.g. Cabbage, peppers)	9
**Sub - Total Count**			**51**
	Starch	Potatoes (as part of stew or potato salad)	6
		Mielies	2
		Maize meal	8
		Rice	8
		Pasta	6
**Sub - Total Count**			**30**
**Total Count**			**111**
Protein + Majorly Starch			
	Protein	Chicken	5
		Red meat	6
		Legumes	4

		Fish	2
		Processed meat	2
**Sub - Total Count**			**19**
	Starch	Potatoes (as part of stew or potato salad)	9
		Maize meal	3
		Rice	5
		Pasta	1
		Samp	2
		Bread	2
**Sub - Total Count**			**22**
**Total Count**			**41**
Only starch			
	Hot chips + bread		3
	Hot chips		1
	Maize meal		2
	Rice + potatoes		1
**Total Count**			**7**
Bread Meals			
	Bread	White	15
		Brown	5
**Sub - Total Count**			**20**
	Fillings	Polony	12
		Russians	2
		Peanutbutter	2
		Burger Patty	2
		Egg	2
**Sub - Total Count**			**20**
**Total Count**			**40**
Special Meals			
	Sundays		6
	Funerals		1
	Birthdays		3
**Total Count**			**10**

Theme 4: Family

S-theme	SS - theme	SSS -theme	Count
Eating together			1
	Not Daily		5
**Total Count**			**6**
Where they eat			
	In front of the TV		4
	At the table		4
	In the bedroom		2
**Total Count**			**10**
What they eat together			
	Meals		3
	Snacks	Chips	2
		Sweets	1
		Other (Peanuts, slangetjies, Mageu)	3
**Total Count**			**9**
Family likes			
	Fruit		2
	Vegetables		7
	Meals		7
	Snacks	Chips	2
		Sweets and chocolates	1
		Other (chicken gizzards,slangetjies, cake)	3
	Fizzy drinks		2
**Total Count**			**24**
Family dislikes			
	Snacks	Chicken feet	1
**Total Count**			**1**

Theme 5: Peer engagement

S-theme	SS - theme	SSS -theme	Count
Eating together			
	Daily		3
	Not daily		3
**Total Count**			**6**
What they eat together			
	Take-aways		1
	Snacks	Chips	2
		Sweets	3
		Fizzy drink	1
	Sandwiches		2
**Total Count**			**9**
Where they eat			
	at school		4
	at each others' houses		2
**Total Count**			**6**
Likes			
	Snacks	Chips	2
		Sweets	1
	Fizzy drinks		1
**Total Count**			**3**

Theme 6: Food preparation

S - theme	SS - theme	Count
Who prepares meals		
	Mother	15
	Father	2
	Learner	5
	Other	7
**Total Count**		**29**
Equipment		
	Two-plate stove	9
	Gas Stove	3
	Braai	2
	Paraffin heater	2
**Total Count**		**16**
Healthy		
	Stir frying	1
	Braai	2
	Stew	8
**Total Count**		**11**
Unhealthy		
	Frying	11
**Total Count**		**11**
